# Association and Interaction Effect of *AGTR1* and *AGTR2* Gene Polymorphisms with Dietary Pattern on Metabolic Risk Factors of Cardiovascular Disease in Malaysian Adults

**DOI:** 10.3390/nu9080853

**Published:** 2017-08-09

**Authors:** Roseline Wai Kuan Yap, Yoshihiro Shidoji, Wai Sum Yap, Motofumi Masaki

**Affiliations:** 1 School of Biosciences, Taylor’s University, 47500 Subang Jaya, Malaysia; 2 Graduate School of Human Health Science, University of Nagasaki, Nagayo-cho, Nagasaki 851-2195, Japan; shidoji@sun.ac.jp (Y.S.); masaki@sun.ac.jp (M.M.); 3 Faculty of Applied Sciences, UCSI University, Taman Connaught, 56000 Kuala Lumpur, Malaysia; wsyap@ucsiuniversity.edu.my

**Keywords:** gene-diet interaction, *AGTR1*, *AGTR2*, rs5186, rs1403543, blood lipids, blood pressure, Malaysian adults

## Abstract

Gene-diet interaction using a multifactorial approach is preferred to study the multiple risk factors of cardiovascular disease (CVD). This study examined the association and gene-diet interaction effects of the angiotensin II type 1 receptor (*AGTR1*) gene (rs5186), and type 2 receptor (*AGTR2*) gene (rs1403543) polymorphisms on metabolic risk factors of CVD in Malaysian adults. CVD parameters (BMI, blood pressure, glycated hemoglobin, total cholesterol (TC), triglycerides, low-density lipoprotein cholesterol, high-density lipoprotein cholesterol (HDL-C), and TC/HDL-C ratio), and constructed dietary patterns “vegetables, fruits, and soy diet” (VFSD), and “rice, egg, and fish diet” (REFD) were obtained from previous studies. Genotyping analysis was performed by real-time PCR using Taqman probes. The subjects were 507 adults (151 Malays; 179 Chinese; and 177 Indians). Significant genetic associations were obtained on blood lipids for rs5186 in Malays and Chinese, and rs1403543 in Chinese females. The significant gene-diet interaction effects after adjusting for potential confounders were: rs5186 × VFSD on blood pressure in Malays (*p* = 0.016), and in Chinese on blood lipids for rs5186 × REFD (*p* = 0.009–0.023), and rs1403543 × VFSD in female subjects (*p* = 0.001–0.011). Malays and Chinese showed higher risk for blood pressure and/or lipids involving rs5186 and rs1403543 SNPs together with gene-diet interactions, but not Indians.

## 1. Introduction

Non-communicable diseases (NCDs) have become a global threat affecting most countries, including Malaysia, an upper-middle-income country. The common NCDs include cardiovascular diseases (CVDs), cancers, respiratory diseases, and diabetes mellitus. The four key metabolic risk factors which increase the risk of NCDs include overweight/obesity, raised blood pressure, hyperglycemia and hyperlipidemia/hypercholesterolemia. The World Health Organization (WHO) has also identified four key modifiable behavioural risk factors of NCDs which are tobacco use, physical activity, diet, and alcohol use [1].

In Malaysia, the current prevalence of total deaths due to NCDs is estimated to be 73% with CVDs being the biggest contributor [2]. The National Health and Morbidity Surveys (NHMS) in Malaysia which provides the most comprehensive health data of adults for 1996, 2006, 2011, and 2015 also showed an increasing trend in all the NCD risk factors. According to the two most recent NHMS in 2011 and 2015, the NCD risk factors which had an increase in the prevalence over the span of four years include diabetes mellitus (from 15.2% to 17.5%), hypercholesterolemia (from 35.1% to 47.7%), and overweight/obesity (from 44.5% to 48%) [2,3]. As for hypertension, there was a slight decrease of 2.4% in the prevalence from 2011 to 2015 [2,3].

The etiology of NCDs is multi-factorial which includes both non-modifiable and modifiable risk factors. Hence, the approach of gene-environment (G × E) or gene-diet interaction which incorporates all possible risk factors could be most appropriate in providing useful information for the prevention and treatment measures of NCDs/CVD. In this study, the approaches of candidate genes and dietary patterns were used to determine the gene-diet interaction effect on metabolic risk factors of CVD. In dietary intake association studies involving chronic diseases, such as CVD, two approaches—single nutrient analysis, and dietary pattern approach (a combination of foods)—are often used. However, the dietary pattern approach is preferred, especially in nutritional epidemiologic studies [4]. Similar studies have also used the dietary pattern approach in gene-diet interaction studies [5,6]. In addition, significant gene-diet interactions were also observed in our previous study [7]. 

The major NCD is CVD; therefore, it is most appropriate to select candidate genes and related polymorphisms in the cardiovascular system. The renin-angiotensin system (RAS) has been known to be related to heart health and angiotensin II type 1 (*AGTR1*) and type 2 receptor (*AGTR2*) in the RAS possess unique counterregulatory function in blood pressure regulation, which is crucial for the prevention of hypertension and CVD. [8]. With that, the angiotensin II type 1 receptor (*AGTR1*) gene and the angiotensin II type 2 receptor (*AGTR2*) gene were selected as the candidate genes in this study.

One criterion in the selection of single nucleotide polymorphisms (SNPs) in this study is the minor allele frequency (MAF) of more than 0.20 in the Asian population. However, due to the lack of information on the MAF of the SNPs in both *AGTR1* and *AGTR2* genes in any of the three main ethnic groups (Malay, Chinese, and Indian) of the Malaysian population, the criterion of MAF used was in reference to other Asian populations, such as Han Chinese and Indian population. Hence, the selected SNPs were rs5186 of *AGTR1* gene which had MAF >0.20 in both Northern [9] and Southern Indians [10,11], and rs1403543 of *AGTR2* gene in the Han Chinese population [12]. The location of the selected SNPs is as follows: rs5186 of *AGTR1* gene in the 3′ untranslated region of *AGTR1* on the chromosome 3 which leads to an adenine (A) to cytosine (C) transversion [13,14]; and rs1403543 of *AGTR2* gene in the intron 1 of the *AGTR2* on the X chromosome [15]. Several studies have also shown significant associations of *AGTR1* gene rs5186 SNP on several types of NCDs such as hypertension [9] and type II diabetes mellitus [10,16], including a systematic meta-analysis with coronary heart disease [17]. As for *AGTR2* gene rs1403543 SNP, significant associations were also reported for hypertension [18], and preeclampsia [19]. 

Due to the growing epidemic of NCDs/CVD in Malaysia, there is definitely a need for research in this area. Based on our literature search, the investigation of genetic associations involving *AGTR1* gene (rs5186) and *AGTR2* gene (rs1403543) polymorphisms has not been reported in the Malaysian population. Hence, the aim of this study is to examine the association of *AGTR1* gene (rs5186) and *AGTR2* gene (rs1403543) SNPs on the metabolic risk factors of CVD, in addition to gene-diet interaction effects with dietary patterns in multi-ethnic Malaysian adults. The findings of this study may provide insights on the possible risks of CVD which could be associated by the selected candidate genes and related SNPs in addition to the effect of gene-diet interactions.

## 2. Materials and Methods

### 2.1. Study Population and Design

The study subjects in this study were Malaysian adults comprised of the three main ethnic groups (Malay, Chinese, and Indian), aged 30–65 years, and residents of urban Klang Valley. A convenience sampling approach was used in this study which relied on voluntary participation of the subjects. The subjects were healthy, not pregnant, and not hospitalized during the recruitment period of the study. The following information pertaining to the subjects were obtained from our previous studies [20,21]: (1) demographics (age, gender, and ethnicity); (2) health (past/presence of common chronic NCDs and on any medications); (3) lifestyle habits (exercise, smoking, and alcohol consumption); (4) dietary intakes using validated food frequency questionnaire; and (5) measured parameters related to the metabolic risk factors of CVD. In order to control for the possible effect of medications use for the common chronic NCDs on the study results, a pre-screening process to exclude subjects on medications was performed during the recruitment stage. In addition, subjects who indicated the use of medications in the standard questionnaire were also excluded from the study. The parameters used in this study were body mass index (BMI) for weight status, blood pressure, and the following biomarkers: glycated hemoglobin (HbA1c); and blood lipids (total cholesterol, triglycerides, low-density lipoprotein cholesterol (LDL-C), high-density lipoprotein cholesterol (HDL-C), and total cholesterol/HDL-C ratio). The methods used for the measured parameters were described in detail in our previous study [20]. This study was approved by the Research Ethics Committee of University of Nagasaki, Japan and UCSI University, Malaysia. All subjects provided written informed consent. 

### 2.2. Dietary Patterns

A validated semi-quantitative food frequency questionnaire described in [20] was used to obtain dietary intake information for each subject. The dietary intake information of each subject were then used to construct major dietary patterns using factor analysis and principal component analysis. The two dietary patterns previously constructed from all subjects were: “vegetables, fruits, and soy diet” (VFSD); and “rice, egg, and fish diet” (REFD) [7]. The dietary pattern of VFSD was derived from high consumption of various types of vegetables (green leafy vegetables, cabbage, cauliflower, Chinese cabbage, and broccoli), fruits (papaya, mango, pineapple, starfruit, and papaya), and soybean curd, while the dietary pattern of REFD was comprised of rice, chicken egg, and fish [7]. Based on the two identified dietary patterns (VFSD and REFD), factor scores were derived for each subject in each dietary pattern. The factor scores were then categorized into tertiles for further analysis on gene-diet interactions.

### 2.3. Genotyping

DNA samples of all subjects were collected using buccal mucosal swabs and were obtained from our previous studies [20,21]. The procedures for DNA extraction and purification were also described previously [20]. A real-time PCR system (StepOne™, AppliedBiosystems, Singapore city, Singapore) was applied in the genotyping analyses for the *AGTR1* gene (rs5186) and the *AGTR2* gene (rs1403543) polymorphisms using Taqman^®^ GTXpress Master Mix (Applied Biosystems, Foster City, CA, USA) and ready-made TaqMan probes (Taqman SNP Genotyping Assays, Applied Biosystems, Foster City, CA, USA). All genotyping procedures were performed according to the protocol described by the manufacturer. 

### 2.4. Statistical Analysis

Statistical analyses were performed using Statistical Package for Social Sciences (SPSS Statistics 20.0, IBM SPSS, Armonk, NY, USA). The normality test of each variable for continuous data was tested using the Kolmogorov-Smirnov test. The following tests depend on the normality of the continuous data for the dependent variables, and also the number of categories for the independent variables were used to determine the genetic associations on the metabolic risk factors of CVD in each ethnic group: Student’s *t*-test; Mann-Whitney test; Kruskal-Wallis test; and the analysis of variance (ANOVA) with a post-hoc test, Dunnett T3 (under the condition of equality of variances not assumed). The results of the significant genetic associations obtained were then used as the reference point to determine gene-diet interactions using two-way ANOVA test adjusted for confounding variables: age; sex; smoking; and physical activity. These confounding variables were selected because there are known risk factors which will influence the metabolic risk factors measured in the present study [22,23]. Data of the confounding variables were obtained from the standard questionnaire. Variables which were presented in categorical data include: sex (male/female), smoking (yes/no); and physical activity (yes/no), while age was in the continuous data. A probability value of <0.05 was set as statistically signficant. 

## 3. Results

### 3.1. Characteristics of the Subjects 

The total number of subjects included in the study was 507 Malaysian adults: 151 Malay (*n* = 39 (26%) males and *n* = 112 (74%) females), 179 Chinese (*n* = 75 (42%) males, and *n* = 104 (58%) females), and 177 Indian (*n* = 40 (23%) males and *n* = 137 (77%) females). Table 1 summarises the physical, biochemical (measured metabolic risk factors of NCDs), and genetic characteristics of the subjects by ethnic group. In terms of gender, there were more females compared to males with significant differences in mean for blood lipids. Females in all ethnic groups had significantly lower mean values for blood triglycerides, and total cholesterol/HDL-C ratio, but higher in mean value for HDL-C compared to the males (*p* < 0.05). In comparison between ethnic groups for blood lipids, Malays had significantly higher mean values for total cholesterol and LDL-C compared to Chinese while Indians had significantly lower mean values for HDL-C compared to Malays and Chinese (Table 1). 

The genotype frequencies for *AGTR1* gene rs5186 SNP in the Malays were: 86.8% of AA (*n* = 131), 12.6% of AC (*n* = 19) and 0.6% of CC (*n* = 1); Chinese: 88.3% of AA (*n* = 158), 11.7% of AC (*n* = 21), and none with the CC genotype; and Indians: 88.1% of AA (*n* = 156), 11.9% of AC (*n* = 21), and also none with the CC genotype. The MAF for Chinese subjects was similar to Han Chinese, while the MAF in other Asian populations, such as the Japanese, was also small, which is 0.03 [24]. As for the *AGTR2* gene rs1403543 SNP, the genotype frequencies of each ethnic group were separated by gender because the *AGTR2* gene is on the X chromosome, therefore, the male subjects will only have one allele. The genotype frequencies for males were Malay: 46.0% of A allele (*n* = 18), and 54.0% of G allele (*n* = 21); Chinese: 53.3% of A allele (*n* = 40), 46.7% of G allele (*n* = 35); and Indians: 60.0% of A allele (*n* = 24) and 40.0% of G allele (*n* = 16) while for females were Malay: 21.4% of AA (*n* = 24), 54.5% of AG (*n* = 61), and 24.1% of GG (*n* = 27); Chinese: 32.7% of AA (*n* = 34), 53.8% of AG (*n* = 56), 13.5% of GG (*n* = 14); and Indians: 21.9% of AA (*n* = 30), 48.9% of AG (*n* = 67), and 29.2% of GG (*n* = 40). The genotypes’ SNP sites for the Malaysian subjects in this study were conformed to the Hardy-Weinberg equilibrium using a web-based tool [25].

### 3.2. Associations of AGTR1 Gene (rs5186) and AGTR2 Gene (rs1403543) Polymorphisms with Metabolic Risk Factors of CVD

The associations of the *AGTR1* gene (rs5186) SNP on all the measured metabolic risk factors of CVD by ethnic group (Malay, Chinese, and Indian subjects) were performed using either Student’s *t*-test or the Mann-Whitney test. Significant associations were only obtained for blood triglycerides in both Malay (Table 2) and Chinese subjects (Table 3) in which the AC + CC genotype for Malays and the AC genotype for Chinese were significantly higher in mean blood triglyceride values compared to the AA genotype subjects. There was no significant association of the *AGTR1* gene (rs5186) SNP on all the measured metabolic risk factors of CVD in the Indian subjects (*p* > 0.05).

The associations of the *AGTR2* gene (rs1403543) SNP on all the measured metabolic risk factors of CVD by ethnic group (Malay, Chinese, and Indian subjects) were only performed in females using one-way ANOVA and the Kruskal–Wallis test. This is due to the lower number of male subjects (Malay: 39; Chinese: 75 and; Indian: 40) compared to the females in this study. In addition, the males are of monoallele, as explained earlier. Significant associations were only obtained for blood LDL-C and total cholesterol/HDL-C ratio levels in Chinese subjects (Table 4). The AA genotype subjects had the highest the mean in blood LDL-C levels, while the AG genotype subjects had the highest mean in total cholesterol/HDL-C ratio. Further analysis using a post-hoc test have shown there was no significant difference in mean LDL-C levels between the three genotypes of the *AGTR2* gene (rs1403543) SNP, while the AG genotype subjects had significantly higher mean total cholesterol/HDL-C compared to the GG subjects (Table 4). However, for the Malay and Indian subjects, we did not obtain any significant genetic associations involving rs1403543 on all metabolic risk factors of CVD (*p* > 0.05).

### 3.3. Gene-Diet Interactions between AGTR1 Gene (rs5186) and AGTR2 Gene (rs1403543) with Dietary Patterns on Metabolic Risk Factors of CVD

The analyses on the gene-diet interaction effects using two-way ANOVA were only performed based on the previous analyses on the genetic associations of the *AGTR1* gene (rs5186) SNP and the *AGTR2* gene (rs1403543) SNP which resulted in significance. In the Malay subjects, there were significant gene-diet interaction effects between rs5186 and VFSD on both systolic (*p* = 0.026; power = 67.6%) and diastolic (*p* = 0.016; power = 73.9%) blood pressures (Table 5). It is shown that the combination of AC + CC genotype of rs5186 and highest tertile of VFSD had the highest mean in both systolic and diastolic blood pressures compared to the other combinations. On the other hand, for the combination of AA genotype and tertiles of VFSD, it is observed that the AA genotype + tertile 1 of VFSD had the highest mean in both systolic and diastolic blood pressures compared to AA genotype + tertile 2 or 3. There was also significant gene-diet interaction effect between rs5186 and REFD on blood triglycerides in Malay subjects. However, this result was not taken into consideration because it violated the assumption that the variances are equal, which is required in the ANOVA test.

In the Chinese subjects, significant gene-diet interaction effects were obtained for rs5186 and REFD on blood total cholesterol (*p* = 0.023; power = 69.9%) and LDL-C (*p* = 0.009; power = 77.8%) (Table 6), and rs1403543 and VFSD on blood total cholesterol (*p* = 0.011; power = 79%) and HDL-C (*p* = 0.001; power = 95.6%) (Table 7). The combination of AC genotype of rs5186 and tertile 3 of REFD had the highest mean in total cholesterol and LDL-C levels compared to the other combinations. It was also observed that, for the combination of the AC genotype with the tertiles of REFD, there was an increasing trend in the mean of total cholesterol and LDL-C from the lowest tertile (tertile 1) to the highest tertile (tertile 3). However, a reverse trend was observed in the AA genotype of rs5186 in which there was a decreasing trend in the mean of total cholesterol and LDL-C from the lowest tertile (tertile 1) to the highest tertile (tertile 3). Lastly, in the Chinese female subjects, it was shown that the means of total cholesterol and HDL-C were highest in the combination of the AA genotype of rs1403543 and tertile 2 of VFSD compared to the other combinations (Table 7). The gene-diet interaction analyses were not performed in the Indian subjects because there were no significant genetic associations on the metabolic risk factors of CVD in the Indian subjects.

## 4. Discussion

The increasing prevalence of NCDs in Malaysia and the associated metabolic risk factors has become a health threat to the population. In addition, the effect of the high healthcare costs associated with these chronic diseases has also created an economic burden to the population. In Malaysia, studies on gene-diet and gene-environment interaction involving NCDs are scarce [26]. Hence, the present study evaluated the genetic associations and gene-diet interactions involving *AGTR1* and *AGTR2* gene polymorphisms in Malaysian adults. Interestingly, we found significant genetic associations and gene-diet interactions of both *AGTR1* and *AGTR2* gene polymorphisms on two key metabolic risk factors of CVD, which are blood pressure and blood lipids.

In the present study on the genetic associations involving *AGTR1* gene (rs5186) SNP, we found that the C allele may be the risk allele for the blood lipid, triglycerides as the mean blood triglycerides for the AC + CC genotype in Malays and AC genotype in Chinese were significantly higher compared to AA genotype subjects. In comparison with other literatures, a similar finding was also obtained in a study among Han Chinese subjects, in which significant association was found in AC + CC genotypes of rs5186 with essential hypertension, and the CC genotype subjects had a higher risk of developing essential hypertension compared to subjects of other genotypes [9]. In addition, the C allele and the AC/CC genotypes were also found to be significantly associated with type II diabetes mellitus and its comorbidity in Asian Indians [10,16] and with coronary heart disease in East Asia populations [17]. In our study however, we failed to obtain any significant genetic associations on all metabolic risk factors of CVD in Indian subjects. Similarly for the rs5186 of the *AGTR1* gene, significant associations were obtained for the rs1403543 SNP in the *AGTR2* gene with blood lipids in our Chinese Malaysian subjects. In the present study, the A allele could be the risk allele for blood lipids but other studies have shown that the G allele was more susceptible to hypertension [18] and preeclampsia [19]. In our investigation on Malay and Indian subjects, we did not obtain any significant genetic associations involving rs1403543 on all metabolic risk factors of CVD.

The significant genetic associations on blood lipids in this study have indicated a possible relationship between the *AGTR1* and *AGTR2* gene polymorphisms with other metabolic risk factors CVD besides blood pressure. This could be explained by the other roles of AGTR1 and AGTR2 which may have an effect on the development of NCDs/CVD. For example, besides the counterregulatory interaction in the regulation of blood pressure whereby AGTR1 plays the role as the vasoconstrictor and AGTR2 as the vasodilator, AGTR1 is also involved in cellular proliferation and growth, while AGTR2 is involved in cell growth and inhibition [27]. 

In our study, significant gene-diet interactions were obtained on blood pressure and selected blood lipids even though the individual genetic associations for these metabolic risk factors of CVD were not obtained. Based on our literature search, we did not find any similar literatures on gene-diet interactions involving *AGTR1* gene (rs5186) and *AGTR2* gene (rs1403543) SNPs. However, there was a study which determined the gene-diet interactions involving the *AGTR1* gene (rs5186) SNP with sodium, potassium, and fluid intakes on renal cell cancer risk [28]. However, this study did not obtain any significant gene-diet interactions, which could also be due to the lack of statistical power [28]. Hence, we speculate that a certain dietary pattern may have an influence on the polymorphism effects of both *AGTR1* gene (rs5186) SNP and *AGTR2* gene (rs1403543) SNP. However, the actual mechanism involving the gene-diet interaction remains unclear, thus warrant further investigation.

There are some limitations in this study which should be noted and were also indicated in the results of our study. The number of male subjects was much lower compared to the female subjects, especially for Malay and Indian subjects, therefore, the analysis on the male subjects could not be performed for *AGTR2* gene (rs1403543) SNP. The sample size may not be sufficient for statistical power because a small MAF was obtained for rs5186 (<0.10) in our subjects. Lastly, our findings were limited as some statistical tests, such as the parametric tests (Student’s *t*-test and one-way ANOVA), and results from two-way ANOVA could not be used due the required pre-requisites (data in normal distribution) or assumptions (equal variance) for specific statistical tests which were not met. 

In summary, significant individual genetic associations and gene-diet interaction effects between the *AGTR1* gene (rs5186) SNP and the *AGTR2* gene (rs1403543) SNP and dietary patterns on blood lipids were obtained particularly in Malay and Chinese Malaysian subjects of our study. The AC + CC genotype in Malay subjects and AC genotype in Chinese subjects of the *AGTR1* gene (rs5186) SNP had higher risks of blood triglycerides compared to the AA genotype subjects. As for the *AGTR2* gene (rs1403543) SNP, the AA genotype and AG genotype in female Chinese subjects had the highest risk for LDL-C and total cholesterol/HDL-C ratio, respectively. In Malays, the combination of AC + CC genotype of rs5186 and the highest tertile of VFSD had the highest risk of blood pressure while, in Chinese, the combination of the AC genotype of rs5186 and the highest tertile of REFD had the highest risk for blood lipids (total cholesterol and LDL-C). Finally, the combination of the AA genotype of rs1403543 and tertile 2 of VFSD had a higher risk of total cholesterol, but a lower risk in HDL-C compared to the other combinations in Chinese female subjects. The Chinese female subjects who had the highest risk for HDL-C was from the combination of AG + GG genotype and tertile 2 of VFSD.

## 5. Conclusions

This may be the first report which showed significant genetic associations and gene-diet interactions involving the *AGTR1* gene (rs5186) SNP and the *AGTR2* gene (rs1403543) SNP on blood pressure and/or blood lipids in Malay and Chinese Malaysian adults, but not in the Indian Malaysian adults. This may imply that Malay and Chinese adults could have higher risk for CVD based on the genotype of *AGTR1* gene (rs5186) and *AGTR2* gene (rs1403543) SNPs in the RAS and also in the interactions with specific dietary patterns. Our findings have also provided insights on the possible relationship of both rs5186 and rs1403543 SNPs with metabolic risk factors of CVD besides chronic NCDs previously reported in other literatures. However, further research involving a larger sample size is recommended to confirm our findings. We also recommend the investigation of gene-diet and gene-environment interactions with other candidate gene polymorphisms in the RAS to curb with the rising epidemic of NCDs/CVD affecting our country, as well as globally.

## Figures and Tables

**Table 1:** Characteristics of the subjects.

Variables	Malay (*n* = 151)	Chinese (*n* = 179)	Indian (*n* = 177)
Age (years)	41 ± 7 ^a^	40 ± 9 ^a^	43 ± 9 ^b^
BMI (kg/m^2^)	25.8 ± 4.71 ^a^	24.2 ± 4.95 ^b^	26.0 ± 4.70 ^a^
SBP (mmHg)	121 ± 15.6	122 ± 14.9	123 ± 15.8
DBP (mmHg)	76.1 ± 10.7	76.7 ± 9.4	76.3 ± 9.5
HbA1c (mmol/mol)	41 ± 0 ^a,b^	40 ± 0 ^a^	45 ± 0 ^b^
Total cholesterol (mmol/L)	5.65 ± 0.92 ^a^	5.31 ± 0.92 ^b^	5.41 ± 0.94 ^b^
Triglycerides (mmol/L)	1.59 ± 0.90	1.81 ± 1.44	1.78 ± 1.08
LDL-C (mmol/L)	3.62 ± 0.87 ^a^	3.08 ± 0.87 ^b^	3.55 ± 0.80 ^a^
HDL-C (mmol/L)	1.35 ± 0.36 ^a^	1.40 ± 0.41 ^a^	1.19 ± 0.31 ^b^
Total cholesterol/HDL-C ratio	4.50 ± 1.53 ^a,b^	4.11 ± 0.92 ^a^	4.80 ± 1.37 ^b^
rs5186 (A allele; C allele)	0.93; 0.07	0.94; 0.06	0.94; 0.06
rs1403543 (A allele; G allele)			
Males	0.46; 0.54	0.53; 0.47	0.60; 0.40
Females	0.49; 0.51	0.60; 0.40	0.49; 0.51

Data are presented in means ± S.D. SBP, systolic blood pressure; DBP, diastolic blood pressure. Analysis performed using one-way ANOVA and the Kruskal–Wallis test with a post-hoc test, Dunnett T3. ^a,b^ different letters indicate significant difference between groups (*p* < 0.05).

**Table 2:** Values of CVD metabolic risk factors according to the genotype of the *AGTR1* gene rs5186 SNP for Malay subjects (*n* = 151).

Variables	rs5186		*p*-Value
	AA (*n* = 131)	AC + CC (*n* = 20)	
BMI (kg/m^2^)	25.9 ± 0.42	25.6 ± 0.88	0.796
SBP (mmHg)	122 ± 1.29	122 ± 3.29	0.818
DBP (mmHg)	76.1 ± 0.94	76.6 ± 2.43	0.830
HbA1c (mmol/mol)	41 ± 0	43 ± 0	0.648
Total cholesterol (mmol/L)	5.61 ± 0.08	5.95 ± 0.27	0.261
* Triglycerides (mmol/L)	1.52 ± 0.08	2.03 ± 0.23	0.012
LDL-C (mmol/L)	3.58 ± 0.07	3.81 ± 0.24	0.579
HDL-C (mmol/L)	1.35 ± 0.03	1.34 ± 0.09	0.971
Total cholesterol/HDL-C ratio	4.46 ± 0.13	4.76 ± 0.38	0.482

Data are presented in means ± S.E. Analysis performed using student *t*-test and Mann-Whitney test, * *p* < 0.05.

**Table 3:** Values of CVD metabolic risk factors according to the genotype of the *AGTR1* gene rs5186 SNP for Chinese subjects (*n* = 179).

Variables	rs5186		*p*-Value
	AA (*n* = 158)	AC (*n* = 21)	
BMI (kg/m^2^)	24.1 ± 0.40	25.2 ± 0.88	0.148
SBP (mmHg)	122 ± 1.19	125 ± 3.28	0.422
DBP (mmHg)	76.6 ± 0.77	77.4 ± 1.69	0.736
HbA1c (mmol/mol)	41 ± 0	43 ± 0	0.748
Total cholesterol (mmol/L)	5.27 ± 0.07	5.57 ± 0.26	0.170
* Triglycerides (mmol/L)	1.74 ± 0.11	2.33 ± 0.36	0.030
LDL-C (mmol/L)	3.06 ± 0.07	3.23 ± 0.26	0.535
HDL-C (mmol/L)	1.42 ± 0.03	1.26 ± 0.08	0.126
Total cholesterol/HDL-C ratio	4.01 ± 0.11	4.82 ± 0.44	0.068

Data are presented in means ± S.E. Analysis performed using Student’s *t*-test and the Mann-Whitney test, * *p* < 0.05.

**Table 4:** Values of CVD metabolic risk factors according to the genotype of the *AGTR2* gene rs1403543 SNP for Chinese female subjects (*n* = 104).

Variables	rs1403543			*p*-Value
	AA (*n* = 34)	AG (*n* = 56)	GG (*n* = 14)	
BMI (kg/m^2^)	23.3 ± 0.73	22.6 ± 0.49	24.4 ± 2.47	0.759
SBP (mmHg)	116 ± 1.95	116 ± 1.70	123 ± 5.81	0.680
DBP (mmHg)	73.8 ± 1.40	74.2 ± 1.21	75.5 ± 3.46	0.845
HbA1c (mmol/mol)	39 ± 0	38 ± 0	41 ± 0	0.396
Total cholesterol (mmol/L)	5.45 ± 0.19	5.12 ± 0.10	4.81 ± 0.23	0.067
Triglycerides (mmol/L)	1.60 ± 0.18	1.31 ± 0.11	1.35 ± 0.20	0.386
* LDL-C (mmol/L)	3.04 ± 0.16	3.02 ± 0.08	2.45 ± 0.23	0.035
HDL-C (mmol/L)	1.65 ± 0.08	1.49 ± 0.05	1.72 ± 0.90	0.084
* Total cholesterol/HDL-C ratio	3.51 ± 0.18 ^a^	3.64 ± 0.15 ^a^	2.91 ± 0.22 ^b^	0.031

Data are presented in means ± S.E. Analysis performed using one-way ANOVA and the Kruskal–Wallis test with pa ost-hoc test, Dunnett T3. * *p* < 0.05; ^a,b^ different letters indicate significant difference between groups.

**Table 5:** Interaction between tertiles of “vegetables, fruits, and soy diet” (VFSD) and genotypes of the *AGTR1* gene rs5186 SNP on metabolic risk factors of CVD in Malay subjects.

Variables	rs5186	VFSD	*n*	Mean ± SE	*p* Interaction
BMI (kg/m^2^)	AA	T1	45	25.8 ± 0.72	0.994
		T2	44	25.6 ± 0.73	
		T3	42	26.5 ± 0.74	
	AC + CC	T1	6	25.2 ± 1.94	
		T2	6	24.8 ± 1.94	
		T3	8	26.0 ± 1.71	
* SBP (mmHg)	AA	T1	45	124 ± 2.25	0.026
		T2	44	120 ± 2.29	
		T3	42	121 ± 2.32	
	AC + CC	T1	6	115 ± 6.07	
		T2	6	114 ± 6.07	
		T3	8	133 ± 5.34	
* DBP (mmHg)	AA	T1	45	77.8 ± 1.58	0.016
		T2	44	74.6 ± 1.60	
		T3	42	75.8 ± 1.63	
	AC + CC	T1	6	71.0 ± 4.25	
		T2	6	71.1 ± 4.25	
		T3	8	85.5 ± 3.74	
HbA1c (mmol/mol)	AA	T1	45	41 ± 0	0.537
		T2	44	41 ± 0	
		T3	42	41 ± 0	
	AC + CC	T1	6	47 ± 0	
		T2	6	43 ± 0	
		T3	8	40 ± 0	
Total cholesterol (mmol/L)	AA	T1	45	5.39 ± 0.15	0.694
		T2	44	5.61 ± 0.15	
		T3	42	5.79 ± 0.16	
	AC + CC	T1	6	6.11 ± 0.41	
		T2	6	5.92 ± 0.41	
		T3	8	6.05 ± 0.36	
Triglycerides (mmol/L)	AA	T1	45	1.45 ± 0.13	0.379
		T2	44	1.37 ± 0.13	
		T3	42	1.71 ± 0.14	
	AC + CC	T1	6	1.79 ± 0.35	
		T2	6	2.41 ± 0.35	
		T3	8	2.19 ± 0.31	
LDL-C (mmol/L)	AA	T1	45	3.42 ± 0.13	0.592
		T2	44	3.59 ± 0.13	
		T3	42	3.72 ± 0.14	
	AC + CC	T1	6	3.97 ± 0.35	
		T2	6	3.59 ± 0.35	
		T3	8	3.94 ± 0.31	
HDL-C (mmol/L)	AA	T1	45	1.33 ± 0.05	0.833
		T2	44	1.37 ± 0.05	
		T3	42	1.33 ± 0.05	
	AC + CC	T1	6	1.40 ± 0.13	
		T2	6	1.43 ± 0.13	
		T3	8	1.29 ± 0.12	
Total cholesterol/HDL-C ratio	AA	T1	45	4.23 ± 0.21	0.826
		T2	44	4.37 ± 0.21	
		T3	42	4.88 ± 0.21	
	AC + CC	T1	6	4.31 ± 0.55	
		T2	6	4.40 ± 0.55	
		T3	8	5.29 ± 0.49	

Data are presented in means ± S.E. Analysis performed using two-way ANOVA adjusted for age, sex, smoking, and physical activity. * *p* < 0.05.

**Table 6:** Interaction between tertiles of “rice, egg, and fish diet” (REFD) and genotypes of the *AGTR1* gene rs5186 SNP on the metabolic risk factors of CVD in Chinese subjects.

Variables	rs5186	REFD	*n*	Mean ± SE	*p* Interaction
BMI (kg/m^2^)	AA	T1	53	24.4 ± 0.70	0.66
		T2	51	24.7 ± 0.68	
		T3	54	23.2 ± 0.67	
	AC	T1	7	23.9 ± 1.83	
		T2	9	26.5 ± 1.62	
		T3	5	25.3 ± 2.16	
SBP (mmHg)	AA	T1	53	121 ± 1.83	0.461
		T2	51	123 ± 1.78	
		T3	54	121 ± 1.77	
	AC	T1	7	130 ± 4.80	
		T2	9	123 ± 4.25	
		T3	5	126 ± 5.67	
DBP (mmHg)	AA	T1	53	76.4 ± 1.29	0.779
		T2	51	77.6 ± 1.26	
		T3	54	75.8 ± 1.25	
	AC	T1	7	79.4 ± 3.38	
		T2	9	77.8 ± 3.00	
		T3	5	75.3 ± 3.99	
HbA1c (mmol/mol)	AA	T1	53	41 ± 0	0.844
		T2	51	39 ± 0	
		T3	54	38 ± 0	
	AC	T1	7	39 ± 0	
		T2	9	40 ± 0	
		T3	5	40 ± 0	
* Total cholesterol (mmol/L)	AA	T1	53	5.37 ± 0.13	0.023
		T2	51	5.31 ± 0.13	
		T3	54	5.14 ± 0.13	
	AC	T1	7	5.16 ± 0.34	
		T2	9	5.43 ± 0.30	
		T3	5	6.42 ± 0.40	
Triglycerides (mmol/L)	AA	T1	53	1.81 ± 0.20	0.39
		T2	51	1.93 ± 0.19	
		T3	54	1.53 ± 0.19	
	AC	T1	7	1.73 ± 0.51	
		T2	9	2.86 ± 0.46	
		T3	5	1.96 ± 0.61	
* LDL-C (mmol/L)	AA	T1	53	3.13 ± 0.12	0.009
		T2	51	3.05 ± 0.12	
		T3	54	2.99 ± 0.12	
	AC	T1	7	2.88 ± 0.32	
		T2	9	2.96 ± 0.29	
		T3	5	4.24 ± 0.38	
HDL-C (mmol/L)	AA	T1	53	1.44 ± 0.05	0.841
		T2	51	1.38 ± 0.05	
		T3	54	1.43 ± 0.05	
	AC	T1	7	1.36 ± 0.13	
		T2	9	1.19 ± 0.12	
		T3	5	1.33 ± 0.16	
Total cholesterol/HDL-C ratio	AA	T1	53	4.13 ± 0.18	0.176
		T2	51	4.11 ± 0.18	
		T3	54	3.82 ± 0.18	
	AC	T1	7	4.13 ± 0.48	
		T2	9	5.03 ± 0.43	
		T3	5	5.23 ± 0.51	

Data are presented in means ± S.E. Analysis performed using two-way ANOVA adjusted for age, sex, smoking, and physical activity. * *p* < 0.05.

**Table 7:** Interaction between tertiles of “vegetables, fruit, and soy diet” (VFSD) and genotypes of the *AGTR2* gene rs1403543 SNP on the metabolic risk factors of CVD in Chinese female subjects.

Variables	rs1403543	VFSD	*n*	Mean ± SE	*p* Interaction
BMI (kg/m^2^)	AA	T1	4	27.1 ± 2.45	0.053
		T2	17	21.4 ± 1.20	
		T3	13	24.9 ± 1.34	
	AG + GG	T1	32	22.8 ± 0.88	
		T2	19	23.6 ± 1.14	
		T3	19	22.5 ± 1.12	
SBP (mmHg)	AA	T1	4	117 ± 6.32	0.987
		T2	17	114 ± 3.10	
		T3	13	115 ± 3.46	
	AG + GG	T1	32	120 ± 2.26	
		T2	19	116 ± 2.93	
		T3	19	116 ± 2.88	
DBP (mmHg)	AA	T1	4	78.2 ± 4.60	0.574
		T2	17	73.3 ± 2.26	
		T3	13	72.4 ± 2.52	
	AG + GG	T1	32	74.4 ± 1.64	
		T2	19	75.4 ± 2.13	
		T3	19	74.1 ± 2.10	
HbA1c (mmol/mol)	AA	T1	4	40 ± 0	0.989
		T2	17	38 ± 0	
		T3	13	39 ± 0	
	AG + GG	T1	32	40 ± 0	
		T2	19	37 ± 0	
		T3	19	37 ± 0	
* Total cholesterol (mmol/L)	AA	T1	4	4.63 ± 0.43	0.011
		T2	17	5.90 ± 0.21	
		T3	13	5.09 ± 0.24	
	AG + GG	T1	32	5.20 ± 0.15	
		T2	19	4.91 ± 0.20	
		T3	19	4.98 ± 0.20	
Triglycerides (mmol/L)	AA	T1	4	1.22 ± 0.44	0.254
		T2	17	1.72 ± 0.21	
		T3	13	1.63 ± 0.24	
	AG + GG	T1	32	1.26 ± 0.16	
		T2	19	1.72 ± 0.20	
		T3	19	0.98 ± 0.20	
LDL-C (mmol/L)	AA	T1	4	2.59 ± 0.39	0.129
		T2	17	3.21 ± 0.19	
		T3	13	2.92 ± 0.22	
	AG + GG	T1	32	3.10 ± 0.14	
		T2	19	2.72 ± 0.18	
		T3	19	2.80 ± 0.18	
* HDL-C (mmol/L)	AA	T1	4	1.42 ± 0.20	0.001
		T2	17	1.86 ± 0.10	
		T3	13	1.43 ± 0.11	
	AG + GG	T1	32	1.55 ± 0.07	
		T2	19	1.39 ± 0.09	
		T3	19	1.69 ± 0.09	
Total cholesterol/HDL-C ratio	AA	T1	4	3.45 ± 0.55	0.217
		T2	17	3.42 ± 0.27	
		T3	13	3.67 ± 0.30	
	AG + GG	T1	32	3.61 ± 0.19	
		T2	19	3.71 ± 0.26	
		T3	19	3.05 ± 0.25	

Data are presented in means ± S.E. Analysis performed using two-way ANOVA adjusted for age, sex, smoking, and physical activity. * *p* < 0.05.
